# Tumor-associated macrophages beyond M1/M2 in NSCLC: spatially organized programs, therapy resistance, and translational targeting

**DOI:** 10.3389/fonc.2026.1960553

**Published:** 2026-09-28

**Authors:** Xiao-Long Wang, Ziheng Jiang, Qianqian Lu, Min Fang

**Affiliations:** 1 School of Life Sciences, Henan University, Kaifeng, Henan, China; 2 Henan Key Laboratory of Synthetic Biology and Biomanufacturing, Henan University, Kaifeng, Henan, China

**Keywords:** immune evasion, immunotherapy, lung cancer, macrophage reprogramming, single-cell RNA sequencing, spatial heterogeneity, spatial transcriptomics, therapeutic resistance

## Abstract

Tumor-associated macrophages (TAMs) are abundant and functionally diverse components of the lung tumor microenvironment (TME) and are associated with therapeutic response and resistance in non-small cell lung cancer (NSCLC). Durable responses to immune checkpoint inhibitors (ICIs) and targeted therapies remain limited, with TAM-associated immune evasion and therapy-refractory microenvironments representing potentially relevant components of this resistance landscape. This narrative review prioritizes evidence from human NSCLC specimens and clinically annotated cohorts, especially from single-cell RNA sequencing (scRNA-seq) and spatial transcriptomics, while integrating relevant mechanistic evidence from NSCLC experimental systems, animal models, and related cancer contexts. Moving beyond the M1/M2 paradigm, we synthesize convergent findings to describe recurring, niche-resolved TAM programs. Across NSCLC, we highlight recurrent *SPP1*-associated, *TREM2*-associated, and C1q/LAM-like macrophage programs as a practical framework for integrating recurrent TAM states across spatial and treatment contexts. We propose an integrative framework in which TAM state dynamics are associated with, and may contribute to, therapy-refractory phenotypes during (i) ICI therapy through immune exclusion, (ii) oncogene-targeted therapy through macrophage-associated bypass signaling and survival plasticity, particularly in EGFR-TKI resistance, and (iii) post-radiochemotherapy microenvironmental remodeling, with potential implications for residual disease ecosystems that require further clinical validation, particularly with respect to recurrence and relapse. TAM program signatures and spatial organization provide a rationale for investigating biomarker-guided stratification and macrophage-directed combination strategies targeting state–niche axes. Future work should prioritize clinically validated, state- and niche-resolved multimodal biomarkers and prospective translational studies to support rational pairing with ICIs and targeted therapies.

## 1 Introduction

Lung cancer remains the leading cause of cancer-related mortality worldwide, and non-small cell lung cancer (NSCLC) accounts for approximately 85% of lung cancer cases (1, 2). Immune checkpoint inhibitors (ICIs), molecularly targeted therapies, radiotherapy, and chemotherapy have improved outcomes for selected patients; however, durable benefit remains limited by primary or acquired treatment failure (3–7). These limitations highlight the need to understand tumor-microenvironmental networks that constrain antitumor immunity, support tumor-cell adaptation, or remodel tissue after treatment.

Tumor-associated macrophages (TAMs) are abundant components of the NSCLC tumor microenvironment and have been implicated in immune regulation, tissue remodeling, metabolic adaptation, and therapeutic response (8, 9). The conventional M1/M2 polarization framework, originally derived largely from cytokine-driven *in vitro* models, does not adequately represent the diversity or context dependence of macrophage states in human tumors (10, 11). Single-cell and spatial profiling instead indicate that TAM phenotypes are shaped by multiple related but non-equivalent dimensions, including developmental origin, local microenvironmental cues, spatial context, and treatment exposure (12–18).

This review adopts an integrative but deliberately cautious framework for TAM biology in NSCLC. We treat *SPP1*-associated, *TREM2*-associated, and C1q/LAM-like designations as operational descriptors rather than fixed, mutually exclusive macrophage subsets. These three programs were selected not as an exhaustive taxonomy, but because they recur across multiple NSCLC datasets and provide a useful framework for integrating macrophage state, spatial context, metabolic adaptation, and treatment-associated phenotypes. We then relate these programs to their state–niche associations, functional implications, and treatment-associated remodeling across immunotherapy, oncogene-targeted therapy, and radiochemotherapy (**Figure 1**).

**Figure 1:**
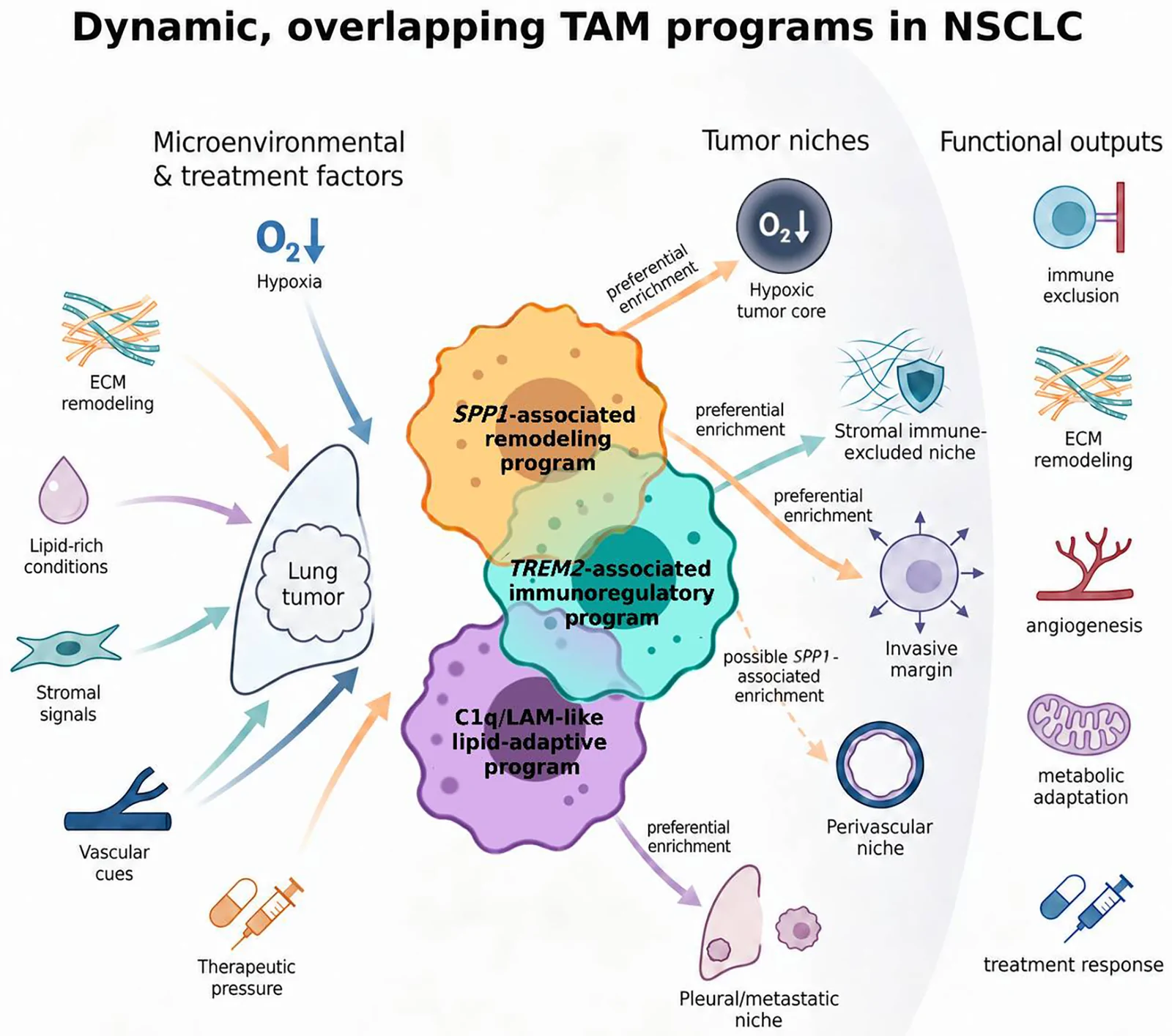
Dynamic, overlapping TAM programs in NSCLC. Tumor-associated macrophages (TAMs) in non-small cell lung cancer (NSCLC) comprise diverse functional programs shaped by hypoxia, extracellular-matrix remodeling, lipid-rich conditions, stromal signals, vascular cues, and therapeutic pressure. These factors contribute to *SPP1*-associated remodeling, *TREM2*-associated immunoregulatory, and C1q/LAM-like lipid-adaptive programs distributed across multiple tumor niches and associated with immune exclusion, extracellular-matrix remodeling, angiogenesis, metabolic adaptation, and treatment response.

Evidence is classified by directness to human NSCLC: Level A1 denotes evidence from human NSCLC specimens or clinically annotated cohorts; Level A2, NSCLC-specific experimental evidence; Level B, biologically plausible evidence from related cancer settings or non-NSCLC models; and Level C, hypothesis-generating concepts requiring NSCLC validation. These levels are used as an interpretive guide rather than formal study-quality ratings. Throughout, observational associations, mechanistic causality, and clinically validated predictive biomarker utility are considered separately.

## 2 TAM lineage origin and high-resolution transcriptional maps: ontogeny and operational programs

TAMs should not be viewed as a single population distributed along a continuous M1–M2 polarization gradient. In NSCLC, their heterogeneity is better considered across related but non-equivalent dimensions: developmental origin, transcriptional and functional state, metabolic program, spatial context, and treatment-associated remodeling. These dimensions interact but are not interchangeable. Developmental origin, local transcriptional state, metabolic adaptation, spatial organization, and treatment exposure should therefore be interpreted as related but distinct features of TAM biology.

### 2.1 Developmental origin and contextual plasticity of TAMs

Lung TAMs can arise from tissue-resident macrophage populations, including alveolar and interstitial macrophages, as well as monocyte-derived macrophages recruited during tumor progression through pathways such as CCL2–CCR2 and CSF1–CSF1R signaling (19–21). These origins may influence baseline localization and responsiveness to local cues, but do not define fixed transcriptional or functional identities.

Single-cell and spatial studies support substantial contextual plasticity in NSCLC TAMs (16, 22–25). Local metabolic conditions, stromal and vascular interactions, immune-cell composition, and treatment exposure may contribute to shaping macrophage programs in NSCLC (16, 22–25). Current spatial datasets more directly resolve functional diversification than lineage assignment; thus, the correspondence between ontogeny, microanatomical location, and state transition remains incompletely defined. Integrated lineage-tracing, spatial-profiling, multiplex-imaging, and longitudinal clinical studies will be useful for clarifying these relationships (26–28). The preferential spatial enrichment and functional relevance of recurrent TAM programs are considered in Section 3 and summarized in **Table 1**.

**Table 1:** Spatial contexts, preferentially enriched TAM programs, and key functions in NSCLC.

Niche	Preferentially enriched TAM program/state*	Key function	Evidence level^†^
Hypoxic/stressed tumor regions	*SPP1*-associated remodeling and metabolically stressed TAM programs	ECM/stromal remodeling and metabolic adaptation	A1/A2; B^a^ (15, 23, 24, 39, 43, 50, 51, 87, 88)
Stromal immune-excluded compartment	*TREM2*-associated lipid-adaptive and immunosuppressive TAM programs	Local immunosuppression and impaired antitumor lymphocyte function	A1/A2^b^ (36–38)
Invasive margin	*SPP1*-associated matrix-remodeling TAM programs	ECM remodeling and invasion-associated tumor-cell plasticity	A1/A2; B^c^ (23, 39–48)
Perivascular niche	Pro-angiogenic TAM programs; possible *SPP1*-associated angiogenic features	Vascular remodeling, angiogenic support, and altered vascular permeability	A1; B/C^d^ (49, 89)
Pleural/metastatic niches	C1q-associated and LAM-like lipid-adaptive TAM programs, including *APOE*/*APOC1*-high states	CD8^+^ T-cell suppression, fatty-acid metabolic adaptation, and metastatic immune dysfunction	A1/A2; B^e^ (52–56)

*State–niche assignments summarize dominant patterns of association and do not imply exclusive localization. ^†^Evidence levels reflect directness to human NSCLC rather than formal study quality: A1, direct human NSCLC evidence; A2, NSCLC-specific experimental evidence; B, indirect evidence from other tumor contexts or models; C, hypothesis-generating evidence requiring NSCLC validation.

^a^Specific lactate–histone lactylation mechanisms remain mainly indirectly supported in NSCLC.

^b^*TREM2*–ICI associations do not establish validated predictive biomarker utility.

^c^Specific mechanotransduction mechanisms remain mainly indirect.

^d^Tie2-related perivascular localization remains indirect or hypothesis-generating in NSCLC.

^e^Broader LAM-like generalization across metastatic niches remains indirect.

### 2.2 Recurrent TAM programs in NSCLC

scRNA-seq and spatial profiling identify recurrent macrophage programs that are more informative than a binary M1/M2 framework (12, 22–25). In this review, *SPP1*-associated, *TREM2*-associated, and C1q/LAM-like programs are highlighted because they recur across selected NSCLC datasets and provide a useful framework for comparing macrophage transcriptional, metabolic, spatial, and functional features (12, 22–25). These programs are partially overlapping and may co-occur with other macrophage-related transcriptional axes (e.g., antigen-presentation-, interferon-responsive-, monocyte-transition-, and resident/proliferative-related programs). Their overlapping functional features are illustrated in **Figure 2**, while key niche-linked functions are summarized in **Table 1** and comparative program-specific features are shown in **Figure 3**. Evidence is interpreted using the A1/A2/B/C scheme; accordingly, human NSCLC associations are treated as direct, whereas many mechanistic interpretations remain incompletely validated in NSCLC.

**Figure 2:**
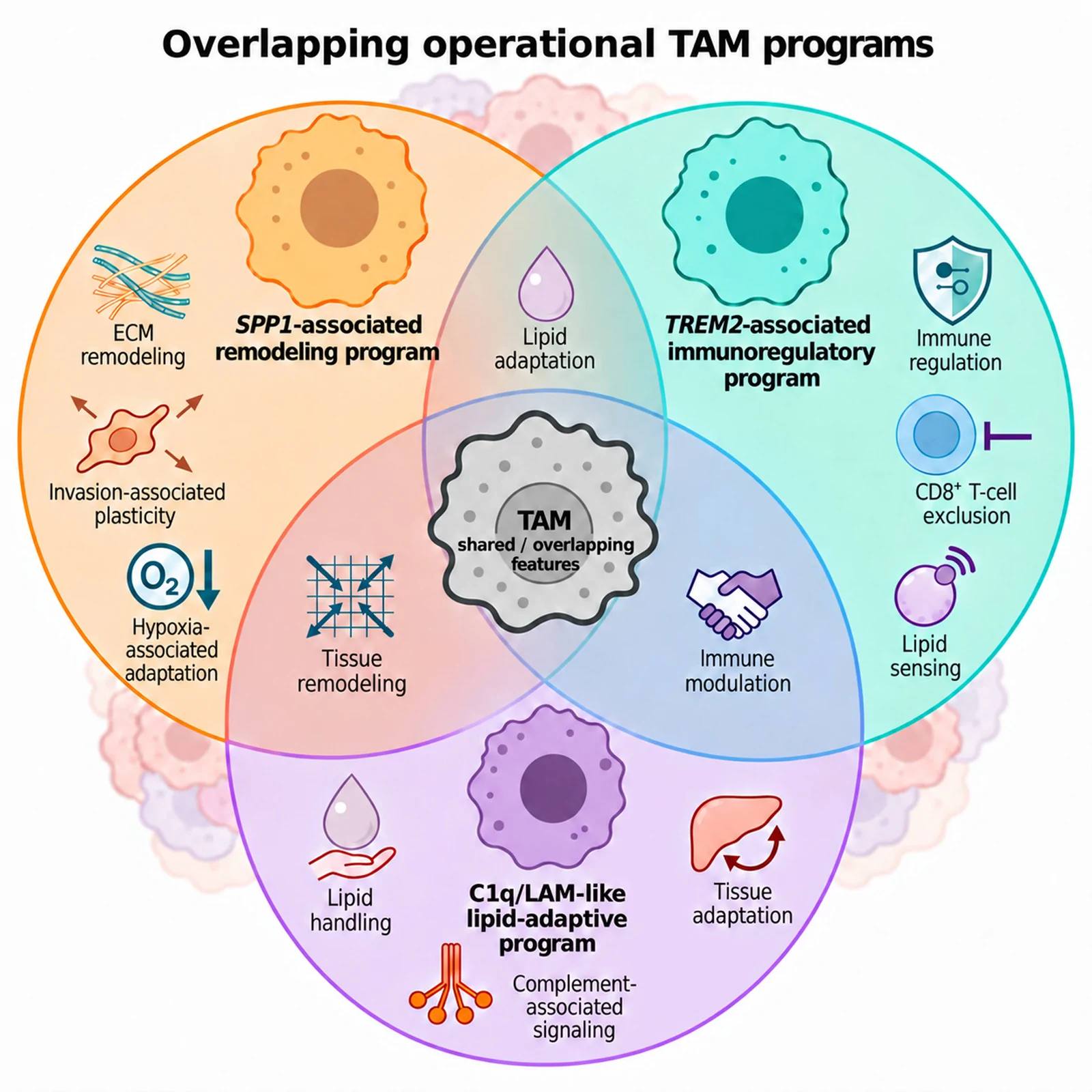
Overlapping functional features of recurrent TAM programs in NSCLC. *SPP1*-associated remodeling, *TREM2*-associated immunoregulatory, and C1q/LAM-like lipid-adaptive programs share selected transcriptional and functional features. Shared regions illustrate common tendencies related to lipid adaptation, tissue remodeling, and immune modulation, while individual macrophages may exhibit features associated with more than one program.

**Figure 3:**
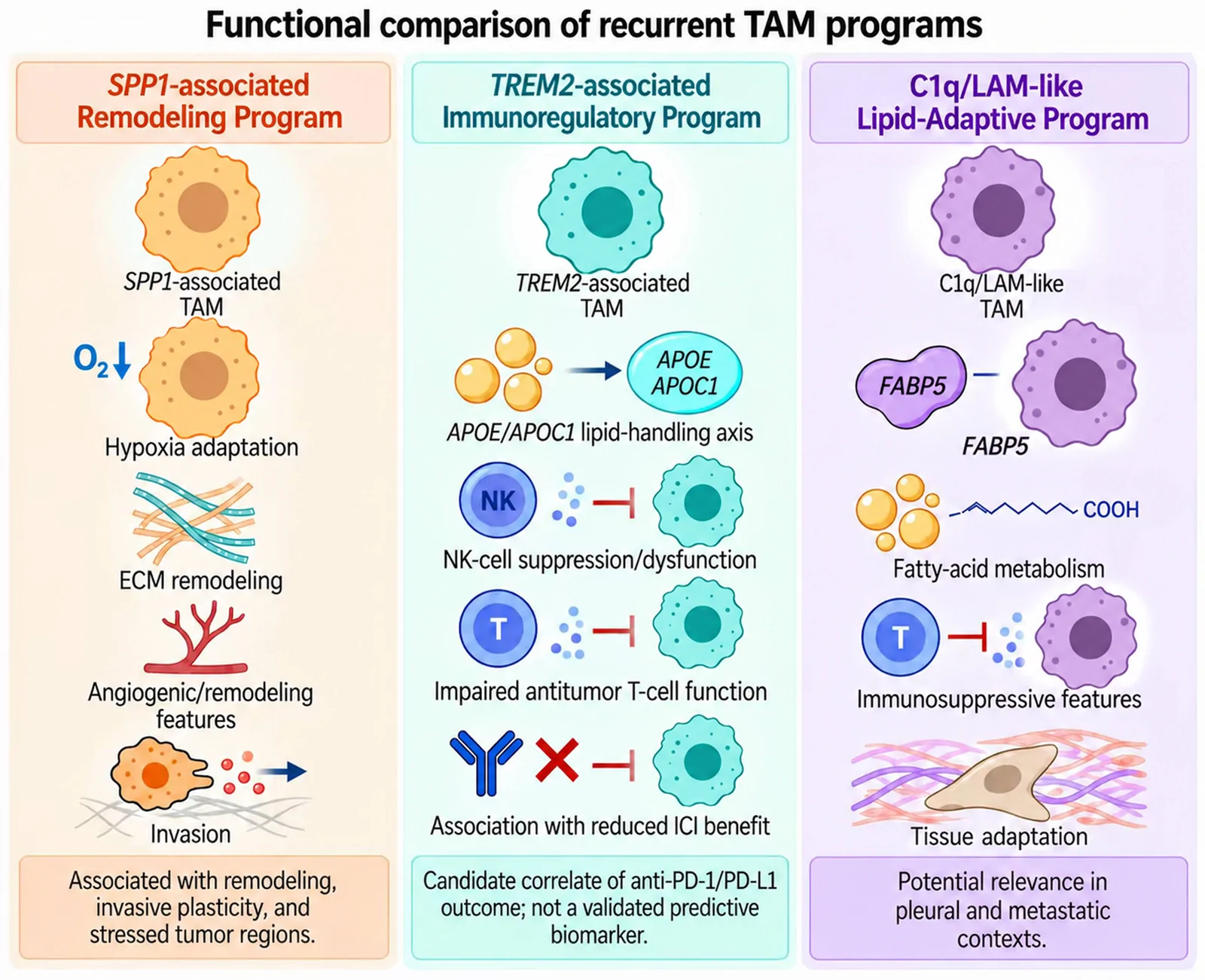
Functional comparison of recurrent TAM programs in NSCLC. *SPP1*-associated remodeling programs are linked to adaptation to hypoxic stress, extracellular-matrix remodeling, angiogenic/remodeling features, and invasion-associated plasticity. *TREM2*-associated immunoregulatory programs show lipid-handling features, including an *APOE*/*APOC1*-associated axis, and are linked to NK-cell dysfunction, impaired antitumor T-cell function, and reduced ICI benefit; their treatment-outcome associations remain candidate correlates rather than validated predictive biomarkers. C1q/LAM-like programs are characterized by lipid-adaptive features, including *FABP5*-associated fatty-acid metabolism, immunosuppressive activity, and tissue adaptation, with potential relevance in pleural and metastatic contexts.

## 3 Spatial organization of TAM programs in NSCLC

Spatial niches provide ecological context for recurrent TAM programs and their functional associations. The following subsections describe state–niche associations and their key functional consequences summarized in **Table 1**, with spatial schematics in **Figure 4** and comparative program features in **Figure 3**.

**Figure 4:**
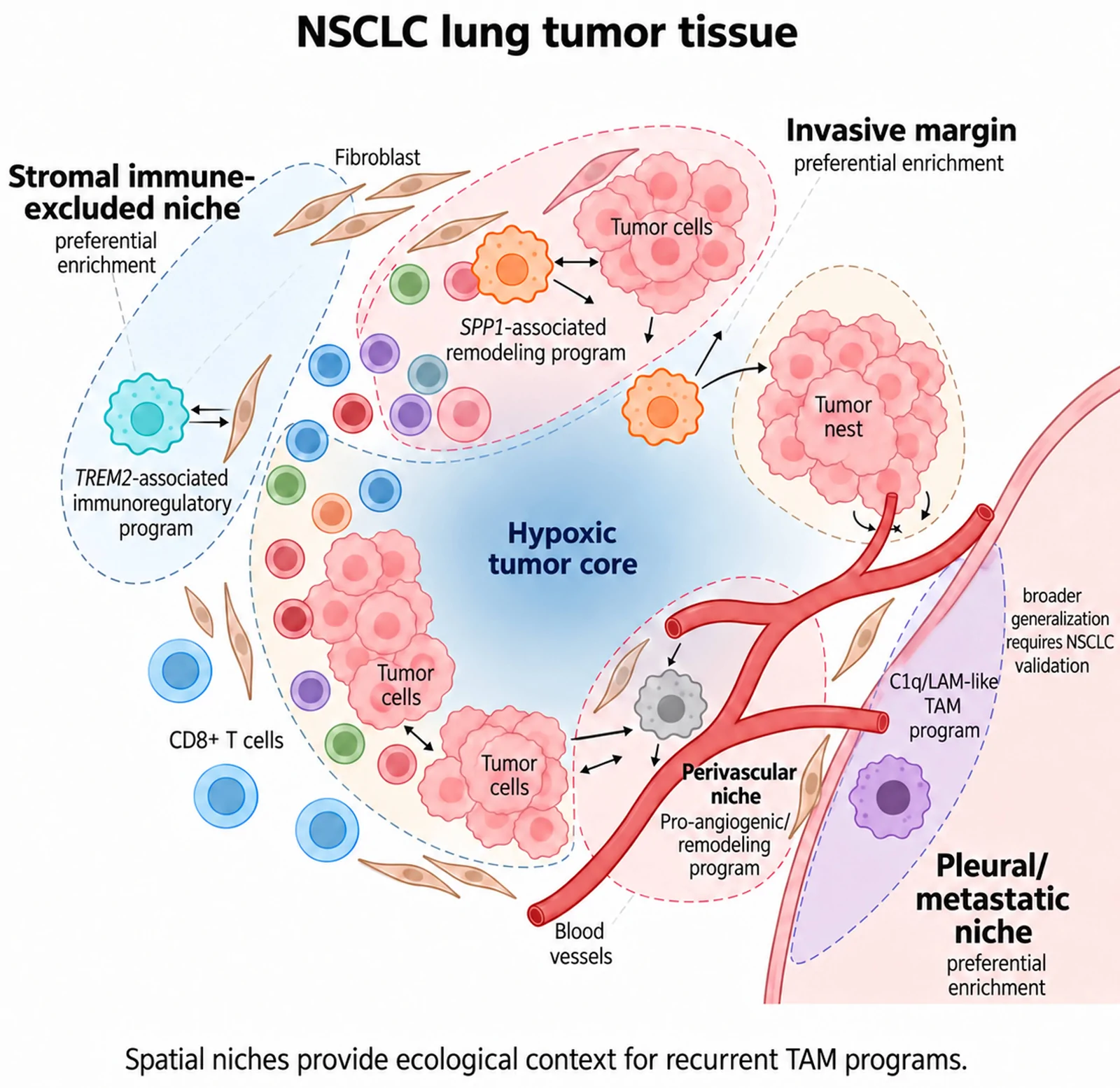
Spatial organization of recurrent TAM programs across NSCLC tissue niches. Recurrent TAM programs show distinct patterns of association across interconnected NSCLC spatial niches. *TREM2*-associated immunoregulatory programs are illustrated in stromal immune-excluded regions, whereas *SPP1*-associated remodeling programs are associated with invasive and matrix-remodeling interfaces. C1q/LAM-like programs are shown in pleural or metastatic contexts. Hypoxic tumor cores and perivascular regions provide additional ecological settings for macrophage adaptation, vascular remodeling, and metabolic stress.

### 3.1 Stromal immune-excluded niches

Stromal immune-excluded compartments can restrict effector lymphocyte access and shape local immunotherapy-related phenotypes. Human NSCLC spatial studies support associations between myeloid-enriched stroma and reduced CD8+ T-cell access/immune suppression (16, 29–34). Mechanistic studies further support the capacity of macrophages to impede CD8+ T-cell access and limit anti-PD-1 efficacy, although this evidence is not specific to human NSCLC spatial cohorts (35). Within these immune-excluded contexts, *TREM2*-associated lipid-adaptive and immunosuppressive macrophage programs have been preferentially observed in selected lung cancer and NSCLC datasets (36–38). The evidence most directly supports the spatial immune-exclusion phenotype itself, whereas specific mechanistic pathways and predictive utility remain to be established for NSCLC.

### 3.2 Invasive and matrix-remodeling fronts

At invasive margins, macrophages are associated with remodeled extracellular matrix and stromal components that can support tumor–stroma communication and invasion-associated remodeling (39–42). NSCLC studies support associations between TAM accumulation, extracellular-matrix remodeling, and invasion-associated tumor-cell plasticity, summarized in **Table 1** (23, 39–48). Mechanistic interpretations involving matrix mechanics, integrin signaling, or YAP/TAZ-mediated mechanotransduction remain largely indirect in NSCLC and should therefore be considered hypothesis-generating rather than established TAM-specific mechanisms.

### 3.3 Hypoxic and perivascular niches

Hypoxic cores and perivascular regions combine metabolic stress with vascular and stromal remodeling. In NSCLC, TAM abundance and associated outcomes correlate with angiogenic/lymphangiogenic programs, while causality is not established (49). Spatial and single-cell datasets suggest enrichment of *SPP1*-associated remodeling programs in metabolically stressed tumor contexts, while the specific spatial relationship between these programs and perivascular niches remains incompletely defined in human NSCLC, as summarized in **Table 1** (39, 50, 51). Direct NSCLC validation of specific mechanistic nodes remains limited.

### 3.4 Pleural and dissemination-associated niches

LAM-like *APOE*/*APOC1*-high macrophage programs have been described in lung-cancer and metastatic contexts, although some supporting evidence derives from non-NSCLC metastatic models and therefore requires disease-specific validation, as summarized in **Table 1** (52–56). These observations support immune and metabolic remodeling in pleural and metastatic settings, but do not establish a uniform mechanism across all metastatic niches. **Figure 4** and **Figure 3** together provide the ecological framework for interpreting treatment resistance and targeting strategies discussed in Sections 4–5.

## 4 TAM-associated treatment resistance and treatment-related remodeling in NSCLC

The clinical relevance of TAMs in NSCLC extends beyond their association with poor prognosis. Across different treatment settings, TAMs are associated with therapy-refractory microenvironmental states and may contribute through distinct mechanisms. During ICI treatment, they are associated with stromal immune exclusion and local immunosuppression. During oncogene-targeted therapy, they may support adaptive, survival-enabling tumor ecosystems. After radiotherapy or chemotherapy, treatment-associated macrophage recruitment and remodeling can influence tissue repair and residual disease environments. However, the strength and directness of evidence vary substantially across these settings. A comparative summary is provided in **Table 2**, and the major treatment contexts are illustrated in **Figure 5**.

**Table 2:** TAM-associated resistance mechanisms and treatment-associated remodeling in NSCLC.

Therapeutic setting	TAM-associated mechanism/remodeling	Key implication	Evidence level*
ICI (PD-1/PD-L1 blockade)	Stromal immune exclusion and suppressive myeloid niches	Associated with reduced benefit from PD-1/PD-L1 blockade; supports evaluation of TAM-directed combination strategies in myeloid-high tumors	A1/A2^a^ (16, 17, 29–34, 51)
ICI (PD-1/PD-L1 blockade)	FcγR-mediated anti-PD-1 antibody sequestration	Biologically plausible mechanism requiring direct validation in NSCLC	B^b^ (90)
ICI (PD-1/PD-L1 blockade)	*TREM2*-associated lipid-adaptive and immunosuppressive TAM programs	Candidate treatment-outcome correlate; not a clinically validated predictive biomarker	A1/A2^c^ (36–38)
EGFR-TKI therapy	Microenvironment-associated bypass signaling, including HGF–MET, with selected macrophage-mediated survival mechanisms	Macrophage/LAM-associated survival and adaptive resistance programs; HGF–MET represents a broader microenvironmental bypass mechanism rather than a uniformly TAM-derived pathway	A1/A2^d^ (55, 57–59)
ALK inhibitor and other targeted therapies	Macrophage- and fibroblast-derived GAS6–AXL signaling	May support pathway-specific macrophage/stromal targeting; evidence should not be generalized across oncogene-driven NSCLC	A2/B^e^ (60)
Radiotherapy	Macrophage recruitment and phenotypic remodeling after treatment	Post-radiotherapy macrophage modulation is testable; a causal role in clinical recurrence or relapse remains unproven	A2/B^f^ (61–64)
Chemotherapy	Treatment-associated macrophage recruitment, tissue-repair programs, and phenotypic remodeling	Post-treatment macrophage modulation is a testable hypothesis; clinical causality for recurrence or relapse remains unproven	A1/A2^g^ (18, 38)

*Evidence levels reflect directness to human NSCLC rather than formal study quality: A1, direct human NSCLC evidence; A2, NSCLC-specific experimental evidence; B, indirect evidence from other tumor contexts or models; C, hypothesis-generating evidence requiring NSCLC validation. Associations with treatment outcome do not alone establish mechanistic causality or predictive biomarker utility.

^a^Spatial immune exclusion and suppressive myeloid ecosystems are supported in human NSCLC, but predictive biomarker utility remains unvalidated.

^b^FcγR-mediated anti-PD-1 sequestration is supported mainly by experimental models.

^c^*TREM2*-associated states correlate with prognosis and anti-PD-1 outcome, but predictive value remains unvalidated.

^d^EGFR-TKI resistance may involve broader microenvironmental mechanisms; HGF should not be assumed to be TAM-derived.

^e^GAS6–AXL signaling is relevant to ALK inhibitor resistance, but GAS6 is not TAM-restricted.

^f^Radiotherapy-induced macrophage recruitment/remodeling is experimentally supported, but its causal role in clinical recurrence remains unproven.

^g^Chemotherapy-associated TAM remodeling is partly supported by combined-treatment data and should not be interpreted as chemotherapy-specific causality.

**Figure 5:**
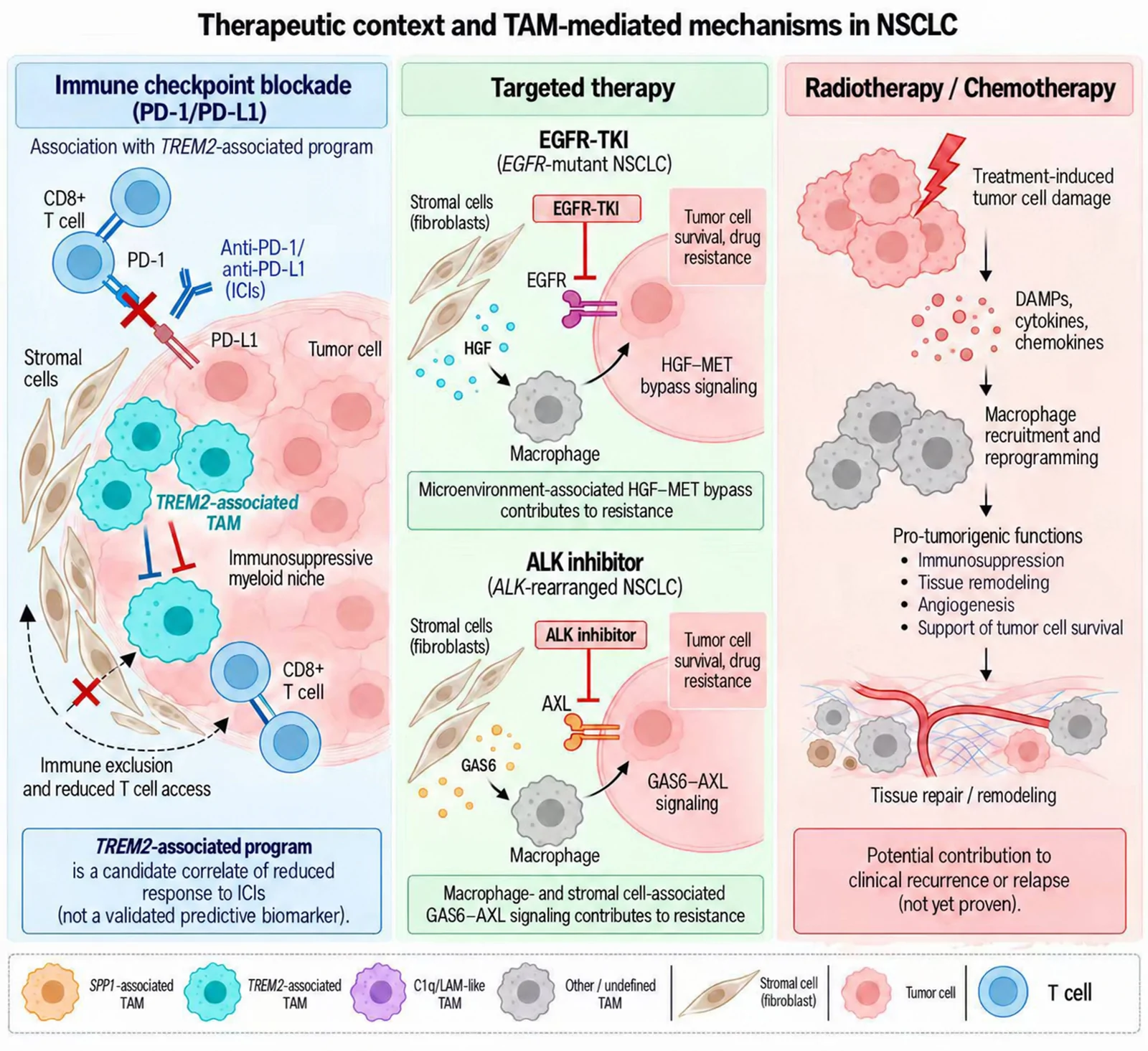
TAM-associated resistance mechanisms and treatment-associated remodeling across major NSCLC treatment settings. TAM-associated mechanisms and microenvironmental remodeling differ across therapeutic contexts. During immune checkpoint inhibition, TAM-rich suppressive myeloid niches and stromal barriers may contribute to immune exclusion and reduced CD8^+^ T-cell access, whereas *TREM2*-associated programs remain candidate treatment-outcome correlates rather than validated predictive biomarkers. In oncogene-driven NSCLC, HGF–MET represents a broader microenvironment-associated bypass mechanism in EGFR-TKI resistance, while macrophage-associated survival support may provide additional resistance signals; TAMs should not be assumed to be the responsible HGF source across all settings. In *ALK*-rearranged disease, macrophage- and stromal-cell-associated GAS6–AXL signaling provides a more direct rationale for microenvironment-mediated survival support. Radiotherapy and chemotherapy can induce tissue damage, macrophage recruitment, and repair-associated remodeling, but a causal role of these macrophage changes in clinical recurrence or relapse remains unproven.

### 4.1 Resistance to ICIs: immune exclusion and local myeloid suppression

Among treatment settings in NSCLC, one of the strongest clinical rationales for TAM involvement concerns ICI response in immune-excluded, myeloid-rich tumor architectures. In human NSCLC, stromal immune exclusion and suppressive myeloid niches are associated with reduced benefit from PD-1/PD-L1 blockade, supporting evaluation of TAM-directed combination strategies in myeloid-high tumors (16, 17, 29–34, 51). In parallel, *TREM2*-associated lipid-adaptive and immunosuppressive TAM programs are associated with unfavorable outcomes and reduced anti–PD-1 benefit; however, these *TREM2*-associated measures remain candidate correlates and have not been clinically validated as predictive biomarkers (36–38). Overall, TAM-linked immune exclusion is better supported as a spatial and clinical association than as proof of a clinically actionable TAM predictive biomarker.

### 4.2 Resistance to targeted therapy in oncogene-driven NSCLC

In selected oncogene-driven settings, macrophage-associated signals may contribute to adaptive, non-genetic, and microenvironment-dependent resistance programs. In *EGFR*-mutant NSCLC, microenvironment-associated bypass signaling, including HGF–MET, and distinct macrophage-associated survival mechanisms have been implicated in resistance to EGFR-TKIs. HGF–MET should be regarded as a broader stromal bypass mechanism rather than a uniformly TAM-derived pathway, whereas macrophage-associated mechanisms include lipid-associated macrophage programs and macrophage-derived extracellular vesicle signaling (55, 57–59). For *ALK*-rearranged NSCLC, macrophage- and fibroblast-derived GAS6–AXL signaling provides a more direct rationale for pathway-specific co-targeting (60). Accordingly, TAM-directed approaches are most plausible when targeted-therapy failure reflects adaptive, state-driven features, whereas broader clinical translation requires prospective validation.

### 4.3 Treatment-associated remodeling after radiotherapy and chemotherapy

Radiotherapy and chemotherapy can induce tissue injury and inflammatory remodeling, which may alter monocyte recruitment and TAM abundance or functional states. Treatment-associated macrophage responses can support debris clearance, matrix/vascular repair, immunoregulation, and inflammatory activation; however, the step from remodeling to clinical recurrence or relapse remains unproven in human NSCLC. Therefore, radiotherapy/chemotherapy-associated TAM changes are best framed as testable treatment-context remodeling processes, with therapeutic vulnerability and timing requiring prospective studies (18, 38, 61–64).

## 5 Translational landscape: clinically relevant strategies for targeting TAMs in NSCLC

The translational goal of TAM-directed therapy is selective modulation of macrophage recruitment, survival, function, or tumor-microenvironment interactions rather than indiscriminate macrophage depletion. Because TAM states are heterogeneous, dynamically shaped by local cues, and spatially organized, the most appropriate intervention can differ by treatment setting, resistance phenotype, and the dominant TAM program. Current modalities range from recruitment/survival blockade and functional reprogramming to phagocytic checkpoint targeting, as well as emerging delivery and engineered-macrophage approaches. Their translational rationale and principal clinical constraints are summarized in **Table 3**, while the treatment-context logic is schematically represented in **Figure 6**.

**Table 3:** Translational strategies for TAM modulation and macrophage-based therapy in NSCLC.

Strategy	Representative target	Biological purpose	Clinical status	Major limitation
Recruitment/survival blockade	CSF1–CSF1R; CCL2–CCR2	Reduce TAM maintenance and monocyte recruitment	Preclinical to early clinical development; no established standard-of-care role in NSCLC	Compensatory myeloid recruitment, limited monotherapy activity, and need for biomarker-guided combinations (19, 20, 59)
Functional reprogramming	STAT6; PI3Kγ; purine-metabolism pathways	Modulate suppressive or repair-associated TAM programs while preserving macrophage viability and potentially enhancing antitumor immune activity	Predominantly preclinical in NSCLC	Heterogeneous responses, uncertain treatment timing, and limited NSCLC-specific validation for some targets (64–66)
Phagocytic checkpoint targeting	CD24; CD47–SIRPα; Siglec-10	Restore macrophage phagocytosis and potentially improve antigen presentation	Clinical maturity varies by target; CD47-directed approaches are more advanced, whereas CD24/Siglec-10 remain emerging	Hematologic toxicity, limited single-agent activity, and incomplete NSCLC-specific evidence (67–69, 91)
Smart delivery platforms	Nanoparticle/RNA-based delivery	Improve spatially localized or macrophage-directed delivery of immunomodulatory cargo and potentially reduce systemic exposure	Predominantly preclinical or early translational	Delivery efficiency, biodistribution, pulmonary safety, and manufacturing scalability remain unresolved (70–73)
Engineered macrophage-modulating platforms	CAR-M	Direct tumor targeting and local immune-microenvironment remodeling	Preclinical foundation; clinical translation remains early, with no established role in NSCLC	Manufacturing feasibility, *in vivo* persistence, tumor trafficking, safety, and lack of NSCLC-specific validation (74)

TAM-directed strategies may influence multiple macrophage programs and microenvironmental compartments; clinical translation requires biomarker-guided selection and NSCLC-specific validation. Clinical experience with CCR2-directed approaches has largely been derived from non-NSCLC settings.

**Figure 6:**
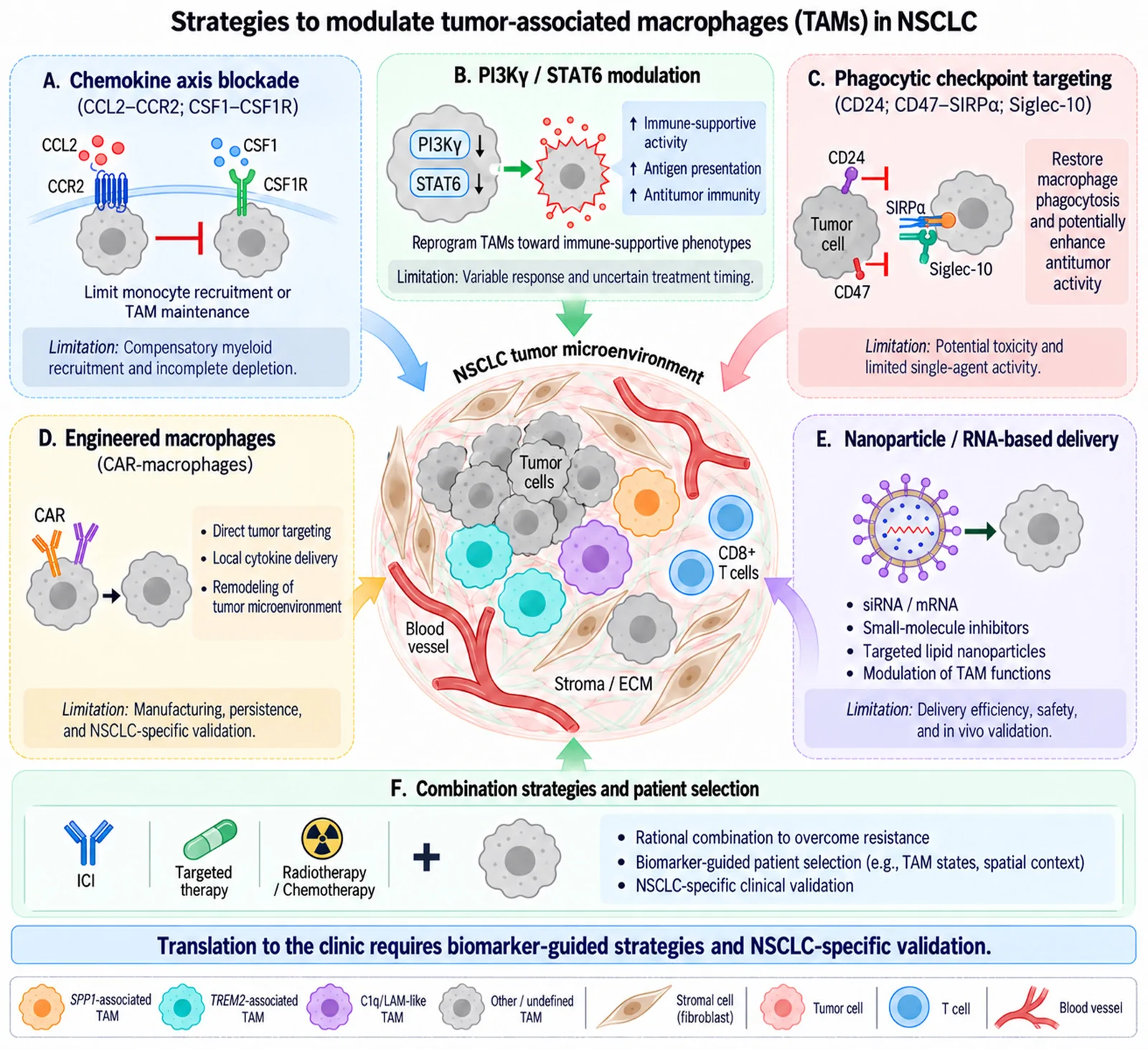
Translational strategies for TAM modulation and macrophage-based therapy in NSCLC. **(A)** Recruitment/survival blockade targeting TAM maintenance and monocyte recruitment through the CCL2–CCR2 and CSF1–CSF1R axes. **(B)** Functional reprogramming approaches targeting macrophage-associated pathways, including PI3Kγ, STAT6, and metabolic programs. **(C)** Phagocytic checkpoint targeting, including CD47–SIRPα, CD24, and Siglec-10 pathways, to enhance macrophage-mediated tumor clearance. **(D)** Smart delivery platforms, including nanoparticle- and RNA-based systems, for localized TAM modulation and reduced systemic exposure. **(E)** Engineered macrophage-based platforms, including CAR-macrophages (CAR-M), for tumor recognition and immune-microenvironment remodeling. **(F)** Biomarker-guided combination strategies integrating TAM modulation with immune checkpoint inhibitors, targeted therapies, or radiochemotherapy. TAM-directed approaches are constrained by heterogeneous macrophage states, compensatory myeloid recruitment, delivery and safety limitations, manufacturing challenges, and the need for NSCLC-specific clinical validation.

A central translational challenge is that macrophage abundance alone is insufficient for patient selection. Macrophages are essential for tissue homeostasis, and TAM-targeting interventions may trigger compensatory myeloid recruitment, impair repair-related functions, or cause systemic toxicity. Accordingly, patient stratification will require state- and niche-resolved biomarkers, together with pharmacodynamic evidence of local on-target modulation in NSCLC (rather than reliance on a single marker-level readout).

### 5.1 Recruitment and survival blockade

Recruitment/survival blockade targets TAM maintenance and/or monocyte influx through the CCL2–CCR2 and CSF1–CSF1R axes (19, 20, 59). These strategies are conceptually relevant when tumors exhibit myeloid-rich or treatment-associated macrophage accumulation and immune-excluded architectures. However, effects are unlikely to be confined to one TAM program; compensatory recruitment via alternative chemokine or myeloid pathways and incomplete elimination of treatment-relevant macrophage states may limit efficacy.

CSF1R inhibition has shown biological activity in preclinical lung-cancer models and may enhance antitumor effects of EGFR-TKI therapy in *EGFR*-mutant lung cancer (59). Clinical development has nevertheless been constrained by target engagement variability and the possibility that residual or newly recruited myeloid populations sustain suppressive or tumor-supportive functions. Therefore, recruitment/survival blockade is best positioned as a combination strategy, with clinical utility depending on demonstrating adequate target engagement and identifying responsive spatial or molecular contexts (**Table 3**; **Figure 6**).

### 5.2 Functional reprogramming

Functional reprogramming aims to reduce suppressive and/or repair-oriented macrophage functions while preserving macrophage viability and tissue-relevant roles. Candidate approaches include modulation of STAT6-dependent signaling, PI3Kγ activity, and purine-metabolism pathways (64–66). These interventions have been evaluated in distinct experimental contexts and should not be regarded as mechanistically equivalent approaches.

A key limitation is that macrophage “states” are rarely defined by a single pathway. Suppressing one program can induce compensatory responses or shift functions differently across niches. Moreover, transcriptomic state shifts inferred from expression profiling do not necessarily demonstrate restored antitumor function *in vivo*. Thus, translational biomarkers must capture spatial and functional consequences of modulation rather than pathway readouts alone. As of now, NSCLC translation for most reprogramming concepts remains preclinical or early, and the most plausible clinical use is in rational combinations—particularly when dominant resistance features involve immune suppression or adaptive stromal support.

### 5.3 Phagocytic checkpoint targeting

Phagocytic checkpoint blockade releases inhibitory “don’t-eat-me” signals to restore macrophage-mediated clearance and potentially improve antigen presentation. The best-characterized phagocytic checkpoint is the CD47–SIRPα axis, whereas CD24 and Siglec-10 represent additional emerging phagocytic checkpoints supported predominantly by preclinical or setting-specific evidence (67–69). The efficacy of these approaches is likely to depend on the local tumor–macrophage interface, including tumor opsonization, antigen availability, and macrophage localization.

Clinical translation has been limited by modest single-agent activity and on-target hematologic toxicities (e.g., anemia), which can constrain dosing. Efficacy may also depend on tumor opsonization, antigen availability, macrophage localization, and the capacity of downstream immune components to respond. Accordingly, phagocytic checkpoint targeting should be evaluated primarily as an immune-activating combination component; clinically validated NSCLC biomarkers for response and pharmacodynamic guidance remain insufficient (**Table 3**; **Figure 6**).

### 5.4 Emerging delivery and engineered-macrophage platforms

RNA-based systems, nanoparticles, and macrophage-targeted nanoplatforms may enable localized modulation of macrophage functions while potentially reducing systemic exposure (70–73). Their key translational value is the ability to influence overlapping TAM programs within defined tissue compartments, rather than globally targeting macrophage subsets.

Engineered macrophages, including CAR-macrophages (CAR-M), add tumor recognition to phagocytosis and immune remodeling (74). However, CAR-M strategies remain predominantly preclinical in NSCLC. Translation challenges include manufacturability, *in vivo* persistence, trafficking into tumor tissue, antigen heterogeneity, and safety. Overall, both delivery platforms and engineered macrophages should be regarded as emerging technologies rather than near-term standards, requiring reproducible manufacturing and clinically relevant evidence of local pharmacodynamic effects and safety (**Table 3**; **Figure 6**).

Overall, TAM-directed therapy in NSCLC should be developed as a biomarker-guided combination strategy informed by the dominant resistance context, spatial organization, and demonstrated target engagement. Clinical benefit will depend on state- and niche-resolved biomarkers, rational treatment timing, and prospective NSCLC validation.

## 6 Conclusion and future roadmap

Advances in single-cell and spatial multi-omics have challenged the traditional binary classification of TAMs into M1 and M2 states. In NSCLC, TAMs are better understood as heterogeneous and dynamically plastic functional programs shaped by developmental origin, local microenvironmental cues, spatial context, and treatment exposure. These programs are associated with, and may contribute to, immune evasion, tissue remodeling, metabolic adaptation, and treatment response, although the strength and directness of evidence vary across these functions. The major translational opportunity therefore lies not in defining a universal TAM taxonomy, but in identifying reproducible state–niche configurations associated with treatment response and amenable to therapeutic modulation. Recurrent *SPP1*-associated, *TREM2*-associated, and C1q/LAM-like programs therefore provide a practical framework for integrating spatial, metabolic, and treatment-associated macrophage biology.

Current evidence supports associations between TAM-rich spatial ecosystems, immune exclusion, and limited treatment benefit (16, 29–34), while stromal/vascular remodeling and metabolic adaptation are supported by additional spatial and experimental studies (23, 39, 49, 51–55, 66). However, the evidence base remains uneven. Human NSCLC studies most directly support spatial and treatment-outcome associations, whereas many proposed mechanisms and therapeutic vulnerabilities remain dependent on experimental systems, indirect evidence, or findings from other tumor types. Accordingly, TAM-associated signatures should not yet be considered clinically validated predictive biomarkers, and treatment-related macrophage remodeling should not be equated with a proven causal role in clinical recurrence or relapse.

### 6.1 Clinical validation of high-resolution spatial interaction atlases

Identifying TAM programs solely on the basis of transcriptomic signatures is insufficient for precise therapeutic intervention. Future studies should use large, well-annotated patient cohorts and integrate spatial transcriptomics, multiplex immunofluorescence, and *in situ* proteomics to define the spatial relationships between recurrent TAM programs, including *SPP1*-associated and *TREM2*-associated programs, and neighboring cancer-associated fibroblasts, endothelial cells, and CD8^+^ T cells within native tumor architecture (16, 34, 39, 40). These analyses should focus on reproducible spatial associations and candidate cell–cell interactions, with functional relationships subsequently tested using appropriate experimental systems.

Integrative multi-omics, machine-learning, and deep-learning approaches may help identify spatially organized TAM programs associated with immunotherapy response by improving feature extraction, data integration, and spatial inference from single-cell and spatial transcriptomic datasets (75, 76). Patient-derived organoid and single-cell approaches may provide complementary systems for testing locally organized immune responses, although their ability to reproduce the full complexity of human tumor architecture requires careful validation (77). Only when these interaction patterns are reproducibly demonstrated in clinically annotated NSCLC specimens, and when they provide information beyond established clinicopathological and molecular variables, will TAM-guided precision strategies have a robust translational foundation (32–34).

### 6.2 Multimodal predictive biomarkers

A major translational challenge is to determine whether TAM-associated spatial and molecular features can improve treatment-response prediction beyond conventional clinical and tumor-intrinsic biomarkers. Baseline peripheral blood single-cell profiling suggests that systemic immune features may provide complementary information on immune checkpoint inhibitor efficacy and toxicity in NSCLC (78). Other molecular and immune-microenvironment biomarkers, including candidate signatures such as *ISG15* and machine-learning–derived immune features, have also been associated with prognosis or immunotherapy-related outcomes in lung cancer, although their incremental value for TAM-guided treatment selection remains unestablished (79, 80). Future biomarker development should prioritize multimodal integration of TAM states, spatial organization, tumor genotype, and treatment exposure rather than reliance on any single macrophage marker or systemic signature.

### 6.3 Senescence-associated microenvironments and emerging intervention windows

Cellular senescence may represent an additional modifier of the lung cancer immune microenvironment, particularly in the context of aging and chronic tissue stress (81, 82). Experimental evidence from *KRAS*-driven lung cancer models indicates that senescent macrophages can contribute to tumor-promoting microenvironmental states, while emerging translational studies suggest that senescence-associated macrophage programs may intersect with chronic inflammatory signaling and tissue remodeling (82–84). However, the extent to which the senescence-associated secretory phenotype shapes TAM states, functional trajectories, and interactions with T cells remains insufficiently defined in a systematic and NSCLC-specific manner. More broadly, macrophage heterogeneity may also be shaped by pre-existing chronic inflammatory or fibrotic lung environments, as suggested by studies of the asthma–lung cancer and pulmonary fibrosis–lung cancer transitions (85, 86). These observations support the concept that chronic tissue context may modify macrophage plasticity, but they should not be interpreted as evidence for a unified senescence-specific TAM state.

These findings justify further investigation of senescence-associated microenvironments as potential modifiers of TAM plasticity, but they do not yet establish a clinically actionable therapeutic axis in NSCLC. Combinations of TAM reprogramming with senolytic or senescence-associated secretory phenotype (SASP)-modulating approaches remain hypothesis-generating and will require NSCLC-specific mechanistic evidence, biomarker-defined patient selection, target-engagement data, and prospective clinical validation.

The next translational priority is to establish state- and niche-resolved biomarkers that can guide context-specific combination therapy. Prospective studies integrating longitudinal tumor sampling, spatial profiling, and treatment-response data should determine when and in which patients TAM-directed interventions can provide additional benefit in combination with ICIs, targeted therapies, or radiochemotherapy. Rather than pursuing indiscriminate macrophage depletion, future approaches should aim to modulate contextually relevant macrophage-associated programs while demonstrating target engagement, acceptable safety, and validated clinical benefit in NSCLC.

## References

[1] Sung H Ferlay J Siegel RL Laversanne M Soerjomataram I Jemal A . Global cancer statistics 2020: GLOBOCAN estimates of incidence and mortality worldwide for 36 cancers in 185 countries. CA Cancer J Clin. (2021) 71:209–49. doi: 10.3322/caac.21660 33538338

[2] Chen Z Fillmore CM Hammerman PS Kim CF Wong KK . Non-small-cell lung cancers: a heterogeneous set of diseases. Nat Rev Cancer. (2014) 14:535–46. doi: 10.1038/nrc3775 25056707 PMC5712844

[3] Borghaei H Paz-Ares L Horn L Spigel DR Steins M Ready NE . Nivolumab versus docetaxel in advanced nonsquamous non-small-cell lung cancer. N Engl J Med. (2015) 373:1627–39. doi: 10.1056/NEJMoa1507643 26412456 PMC5705936

[4] Brahmer J Reckamp KL Baas P Crinò L Eberhardt WEE Poddubskaya E . Nivolumab versus docetaxel in advanced squamous-cell non-small-cell lung cancer. N Engl J Med. (2015) 373:123–35. doi: 10.1056/NEJMoa1504627 26028407 PMC4681400

[5] Schoenfeld AJ Hellmann MD . Acquired resistance to immune checkpoint inhibitors. Cancer Cell. (2020) 37:443–55. doi: 10.1016/j.ccell.2020.03.017 32289269 PMC7182070

[6] Yu J Kong X Feng Y . Tumor microenvironment-driven resistance to immunotherapy in non-small cell lung cancer: strategies for cold-to-hot tumor transformation. Cancer Drug Resist. (2025) 8:21. doi: 10.20517/cdr.2025.14 40342732 PMC12059482

[7] Yan Y Sun D Hu J Chen Y Sun L Yu H . Multi-omic profiling highlights factors associated with resistance to immuno-chemotherapy in non-small-cell lung cancer. Nat Genet. (2025) 57:126–39. doi: 10.1038/s41588-024-01998-y 39658657

[8] Pittet MJ Michielin O Migliorini D . Clinical relevance of tumour-associated macrophages. Nat Rev Clin Oncol. (2022) 19:402–21. doi: 10.1038/s41571-022-00620-6 35354979

[9] Lavin Y Kobayashi S Leader A Amir EAD Elefant N Bigenwald C . Innate immune landscape in early lung adenocarcinoma by paired single-cell analyses. Cell. (2017) 169:750–65:e17. doi: 10.1016/j.cell.2017.04.014 28475900 PMC5737939

[10] Mantovani A Sica A Sozzani S Allavena P Vecchi A Locati M . The chemokine system in diverse forms of macrophage activation and polarization. Trends Immunol. (2004) 25:677–86. doi: 10.1016/j.it.2004.09.015 15530839

[11] Murray PJ Allen JE Biswas SK Fisher EA Gilroy DW Goerdt S . Macrophage activation and polarization: nomenclature and experimental guidelines. Immunity. (2014) 41:14–20. doi: 10.1016/j.immuni.2014.06.008 25035950 PMC4123412

[12] Zilionis R Engblom C Pfirschke C Savova V Zemmour D Saatcioglu HD . Single-cell transcriptomics of human and mouse lung cancers reveals conserved myeloid populations across individuals and species. Immunity. (2019) 50:1317–34:e10. doi: 10.1016/j.immuni.2019.03.009 30979687 PMC6620049

[13] Casanova-Acebes M Dalla E Leader AM LeBerichel J Nikolic J Morales BM . Tissue-resident macrophages provide a pro-tumorigenic niche to early NSCLC cells. Nature. (2021) 595:578–84. doi: 10.1038/s41586-021-03651-8 34135508 PMC8923521

[14] Vitale I Manic G Coussens LM Kroemer G Galluzzi L . Macrophages and metabolism in the tumor microenvironment. Cell Metab. (2019) 30:36–50. doi: 10.1016/j.cmet.2019.06.001 31269428

[15] Leader AM Grout JA Maier BB Nabet BY Park MD Tabachnikova A . Single-cell analysis of human non-small cell lung cancer lesions refines tumor classification and patient stratification. Cancer Cell. (2021) 39:1594–609:e12. doi: 10.1016/j.ccell.2021.10.009 34767762 PMC8728963

[16] Enfield KSS Colliver E Lee C Magness A Moore DA Sivakumar M . Spatial architecture of myeloid and T cells orchestrates immune evasion and clinical outcome in lung cancer. Cancer Discov. (2024) 14:1018–47. doi: 10.1158/2159-8290.CD-23-1380 38581685 PMC11145179

[17] Chen JH Nieman LT Spurrell M Jorgji V Elmelech L Richieri P . Human lung cancer harbors spatially organized stem-immunity hubs associated with response to immunotherapy. Nat Immunol. (2024) 25:644–58. doi: 10.1038/s41590-024-01792-2 38503922 PMC12096941

[18] Cui X Liu S Song H Xu J Sun Y . Single-cell and spatial transcriptomic analyses revealing tumor microenvironment remodeling after neoadjuvant chemoimmunotherapy in non-small cell lung cancer. Mol Cancer. (2025) 24:111. doi: 10.1186/s12943-025-02287-w 40205583 PMC11980172

[19] Cannarile MA Weisser M Jacob W Jegg AM Ries CH Rüttinger D . Colony-stimulating factor 1 receptor (CSF1R) inhibitors in cancer therapy. J Immunother Cancer. (2017) 5:53. doi: 10.1186/s40425-017-0257-y 28716061 PMC5514481

[20] Nywening TM Wang-Gillam A Sanford DE Belt BA Panni RZ Cusworth BM . Targeting tumour-associated macrophages with CCR2 inhibition in combination with FOLFIRINOX in patients with borderline resectable and locally advanced pancreatic cancer: a single-centre, open-label, dose-finding, non-randomised, phase 1b trial. Lancet Oncol. (2016) 17:651–62. doi: 10.1016/S1470-2045(16)00078-4 27055731 PMC5407285

[21] Ginhoux F Guilliams M . Tissue-resident macrophage ontogeny and homeostasis. Immunity. (2016) 44:439–49. doi: 10.1016/j.immuni.2016.02.024 26982352

[22] Yang Q Zhang H Wei T Lin A Sun Y Luo P . Single-cell RNA sequencing reveals the heterogeneity of tumor-associated macrophage in non-small cell lung cancer and differences between sexes. Front Immunol. (2021) 12:756722. doi: 10.3389/fimmu.2021.756722 34804043 PMC8602907

[23] Zhu P Liu Z Husain H Wang Q Zhou Y . Single-cell and spatial analysis reveals macrophage-T cell crosstalk in non-small cell lung cancer immunosuppression. Transl Lung Cancer Res. (2025) 14:4002–20. doi: 10.21037/tlcr-2025-912 41132964 PMC12541844

[24] De Zuani M Xue H Park JS Dentro SC Seferbekova Z Tessier J . Single-cell and spatial transcriptomics analysis of non-small cell lung cancer. Nat Commun. (2024) 15:4388. doi: 10.1038/s41467-024-48700-8 38782901 PMC11116453

[25] DeNardo DG Ruffell B . Macrophages as regulators of tumour immunity and immunotherapy. Nat Rev Immunol. (2019) 19:369–82. doi: 10.1038/s41577-019-0127-6 30718830 PMC7339861

[26] Dizdarević S Wiegandt R Weigert A Stiewe T Eul B Guenther S . Divergent CD45+ immune landscapes shape the lung tumor microenvironment. Front Immunol. (2026) 17:1765833. doi: 10.3389/fimmu.2026.1765833 41737220 PMC12926470

[27] Dong Y Fei X Jiang M Guo H Zhang W Chang X . Decoding the role of macrophage LAP3 in lung cancer: integration of single-cell technologies and machine learning reveals an orchestrating immunometabolic circuit at the tumor-epithelial interface. Front Immunol. (2026) 17:1749190. doi: 10.3389/fimmu.2026.1749190 41836418 PMC12982070

[28] Duval F Lourenco J Hicham M Boivin G Guichard A Wyser-Rmili C . Trajectories of macrophage ontogeny and reprogramming in cancer. iScience. (2025) 28:112498. doi: 10.1016/j.isci.2025.112498 40475851 PMC12139442

[29] Kim J Yong SH Jang G Kim Y Park R Koh HH . Spatial profiling of non-small cell lung cancer provides insights into tumorigenesis and immunotherapy response. Commun Biol. (2024) 7:930. doi: 10.1038/s42003-024-06568-w 39095464 PMC11297140

[30] Larroquette M Guegan JP Besse B Cousin S Brunet M Le Moulec S . Spatial transcriptomics of macrophage infiltration in non-small cell lung cancer reveals determinants of sensitivity and resistance to anti-PD1/PD-L1 antibodies. J Immunother Cancer. (2022) 10:e003890. doi: 10.1136/jitc-2021-003890 35618288 PMC9125754

[31] Fan G Xie T Tang L Li L Han X Shi Y . The co-location of CD14+APOE+ cells and MMP7+ tumour cells contributed to worse immunotherapy response in non-small cell lung cancer. Clin Transl Med. (2024) 14:e70009. doi: 10.1002/ctm2.70009 39187937 PMC11347392

[32] Zugazagoitia J Gupta S Liu Y Fuhrman K Gettinger S Herbst RS . Biomarkers associated with beneficial PD-1 checkpoint blockade in non-small cell lung cancer identified using high-plex digital spatial profiling. Clin Cancer Res. (2020) 26:4360–8. doi: 10.1158/1078-0432.CCR-20-0175 32253229 PMC7442721

[33] Moutafi M Martinez-Morilla S Divakar P Vathiotis I Gavrielatou N Aung TN . Discovery of biomarkers of resistance to immune checkpoint blockade in NSCLC using high-plex digital spatial profiling. J Thorac Oncol. (2022) 17:991–1001. doi: 10.1016/j.jtho.2022.04.009 35490853 PMC9356986

[34] Aung TN Monkman J Warrell J Vathiotis I Bates KM Gavrielatou N . Spatial signatures for predicting immunotherapy outcomes using multi-omics in non-small cell lung cancer. Nat Genet. (2025) 57:2482–93. doi: 10.1038/s41588-025-02351-7 41073787 PMC12513832

[35] Peranzoni E Lemoine J Vimeux L Feuillet V Barrin S Kantari-Mimoun C . Macrophages impede CD8 T cells from reaching tumor cells and limit the efficacy of anti-PD-1 treatment. Proc Natl Acad Sci USA. (2018) 115:E4041–50. doi: 10.1073/pnas.1720948115 29632196 PMC5924916

[36] Park MD Reyes-Torres I LeBerichel J Hamon P LaMarche NM Hegde S . TREM2 macrophages drive NK cell paucity and dysfunction in lung cancer. Nat Immunol. (2023) 24:792–801. doi: 10.1038/s41590-023-01475-4 37081148 PMC11088947

[37] Zhang H Liu Z Wen H Guo Y Xu F Zhu Q . Immunosuppressive TREM2+ macrophages are associated with undesirable prognosis and responses to anti-PD-1 immunotherapy in non-small cell lung cancer. Cancer Immunol Immunother. (2022) 71:2511–22. doi: 10.1007/s00262-022-03173-w 35278107 PMC10991123

[38] Cha YJ Lee EH Kim CY Choi YJ Park MK Lee SH . Anticancer treatment influences TREM2 in tumor-associated macrophages in lung cancer. Cancer Res Treat. (2026) 58:465–80. doi: 10.4143/crt.2024.1245 40575951 PMC13093023

[39] Xiao M Deng Y Guo H Ren Z He Y Ren X . Single-cell and spatial transcriptomics profile the interaction of SPP1+ macrophages and FAP+ fibroblasts in non-small cell lung cancer. Transl Lung Cancer Res. (2025) 14:2646–69. doi: 10.21037/tlcr-2025-244 40799444 PMC12337035

[40] Chen C Guo Q Liu Y Hou Q Liao M Guo Y . Single-cell and spatial transcriptomics reveal POSTN+ cancer-associated fibroblasts correlated with immune suppression and tumour progression in non-small cell lung cancer. Clin Transl Med. (2023) 13:e1515. doi: 10.1002/ctm2.1515 38115703 PMC10731139

[41] Afik R Zigmond E Vugman M Klepfish M Shimshoni E Pasmanik-Chor M . Tumor macrophages are pivotal constructors of tumor collagenous matrix. J Exp Med. (2016) 213:2315–31. doi: 10.1084/jem.20151193 27697834 PMC5068227

[42] Levental KR Yu H Kass L Lakins JN Egeblad M Erler JT . Matrix crosslinking forces tumor progression by enhancing integrin signaling. Cell. (2009) 139:891–906. doi: 10.1016/j.cell.2009.10.027 19931152 PMC2788004

[43] Yang YE Lin YA Ling LL Kuo IY Kuo WT Liu H . Rab37-mediated OPN secretion enriches SPP1+ macrophages through autocrine-paracrine signaling to drive lung tumor progression. Oncogenesis. (2026) 15:4. doi: 10.1038/s41389-026-00596-3 41535255 PMC12823564

[44] Liu Z Wang X Wang Y Zhang G . Integration analysis of single-cell transcriptome reveals SPP1+ and TFF3+ macrophage subsets contributing to the brain metastasis from lung cancer. Transl Cancer Res. (2025) 14:5680–93. doi: 10.21037/tcr-2024-2489 41158243 PMC12554456

[45] Almgrami RT Zhang T Zhao Q You M Liu J Zhang Y . Single-cell transcriptomic analyses provide insights into SPP1+ TAM-mediated immune suppression and CD8+ T cell dysfunction in lung cancer. Cancer Immunol Immunother. (2025) 74:319. doi: 10.1007/s00262-025-04180-3 41021043 PMC12480184

[46] Dupont S Morsut L Aragona M Enzo E Giulitti S Cordenonsi M . Role of YAP/TAZ in mechanotransduction. Nature. (2011) 474:179–83. doi: 10.1038/nature10137 21654799

[47] Zhang S Che D Yang F Chi C Meng H Shen J . Tumor-associated macrophages promote tumor metastasis via the TGF-β/SOX9 axis in non-small cell lung cancer. Oncotarget. (2017) 8:99801–15. doi: 10.18632/oncotarget.21068 29245941 PMC5725132

[48] Guo Z Song J Hao J Zhao H Du X Li E . M2 macrophages promote NSCLC metastasis by upregulating CRYAB. Cell Death Dis. (2019) 10:377. doi: 10.1038/s41419-019-1618-x 31097690 PMC6522541

[49] Hwang I Kim JW Ylaya K Chung EJ Kitano H Perry C . Tumor-associated macrophage, angiogenesis and lymphangiogenesis markers predict prognosis of non-small cell lung cancer patients. J Transl Med. (2020) 18:443. doi: 10.1186/s12967-020-02618-z 33228719 PMC7686699

[50] Wang L Chen B He J Lin C Shen W Lv W . ZFP36L1 promotes non-small cell lung cancer progression under hypoxia by modulating CXCL9:SPP1 polarity: a single-cell transcriptomic study. Clin Transl Med. (2026) 16:e70642. doi: 10.1002/ctm2.70642 41906630 PMC13140207

[51] Wang L Chu H Chen D Wei Y Jia J Li L . SLC2A1+ tumour-associated macrophages spatially control CD8+ T cell function and drive resistance to immunotherapy in non-small-cell lung cancer. Nat Cell Biol. (2026) 28:338–48. doi: 10.1038/s41556-025-01840-5 41501177 PMC12904792

[52] Zhang S Peng W Wang H Xiang X Ye L Wei X . C1q+ tumor-associated macrophages contribute to immunosuppression through fatty acid metabolic reprogramming in malignant pleural effusion. J Immunother Cancer. (2023) 11:e007441. doi: 10.1136/jitc-2023-007441 37604643 PMC10445384

[53] Huggins DN LaRue RS Wang Y Knutson TP Xu Y Williams JW . Characterizing macrophage diversity in metastasis-bearing lungs reveals a lipid-associated macrophage subset. Cancer Res. (2021) 81:5284–95. doi: 10.1158/0008-5472.CAN-21-0101 34389631 PMC8530952

[54] Wu YY Hsu YL Huang YC Su YC Wu KL Chang CY . Characterization of the pleural microenvironment niche and cancer transition using single-cell RNA sequencing in EGFR-mutated lung cancer. Theranostics. (2023) 13:4412–29. doi: 10.7150/thno.85084 37649596 PMC10465223

[55] Li YS Lai WP Yin K Zheng MM Tu HY Guo WB . Lipid-associated macrophages for osimertinib resistance and leptomeningeal metastases in NSCLC. Cell Rep. (2024) 43:114613. doi: 10.1016/j.celrep.2024.114613 39116206

[56] Zhou C Shan S Wen L Liu D Shan C Jin X . Immunological and pathological characteristics of brain parenchymal and leptomeningeal metastases from non-small cell lung cancer. Cell Discov. (2025) 11:72. doi: 10.1038/s41421-025-00828-7 40883281 PMC12397330

[57] Yano S Wang W Li Q Matsumoto K Sakurama H Nakamura T . Hepatocyte growth factor induces gefitinib resistance of lung adenocarcinoma with epidermal growth factor receptor-activating mutations. Cancer Res. (2008) 68:9479–87. doi: 10.1158/0008-5472.CAN-08-1643 19010923

[58] Wan X Xie B Sun H Gu W Wang C Deng Q . Exosomes derived from M2 type tumor-associated macrophages promote osimertinib resistance in non-small cell lung cancer through MSTRG.292666.16-miR-6836-5p-MAPK8IP3 axis. Cancer Cell Int. (2022) 22:83. doi: 10.1186/s12935-022-02509-x 35168607 PMC8845243

[59] Okawa S Tomida S Kuribayashi T Nishimura J Nakasuka T Hirabae A . Colony-stimulating factor-1 receptor inhibitor augments osimertinib-induced antitumor immunity via suppression of macrophages in lung cancer harboring EGFR mutation. Mol Cancer Ther. (2025) 24:1763–74. doi: 10.1158/1535-7163.MCT-25-0002 40439218

[60] Utsumi T Mizuta H Seto Y Uchibori K Nishio M Okamoto I . AXL-mediated drug resistance in ALK-rearranged NSCLC enhanced by GAS6 from macrophages and MMP11-positive fibroblasts. Cancer Sci. (2025) 116:1034–47. doi: 10.1111/cas.70006 39904499 PMC11967273

[61] Yang H Lei Z He J Zhang L Lai T Zhou L . Single-cell RNA sequencing reveals recruitment of the M2-like CCL8high macrophages in Lewis lung carcinoma-bearing mice following hypofractionated radiotherapy. J Transl Med. (2024) 22:306. doi: 10.1186/s12967-024-05118-6 38528587 PMC10964592

[62] Shen M Chen Y Xu L Zhu R Xue X Tsai Y . Increased infiltration of macrophages to radioresistant lung cancer cells contributes to the development of the additional resistance of tumor cells to the cytotoxic effects of NK cells. Int J Oncol. (2018) 53:317–28. doi: 10.3892/ijo.2018.4394 29750425

[63] Li J Bai M Jia W Zhai X Wang M Yu J . Irradiated tumor cell-released microparticles enhance the therapeutic efficacy of PD-1 inhibitors by promoting M1-TAMs polarization in NSCLC brain metastases. Cancer Lett. (2024) 598:217133. doi: 10.1016/j.canlet.2024.217133 39079563

[64] He K Barsoumian HB Puebla-Osorio N Hu Y Sezen D Wasley MD . Inhibition of STAT6 with antisense oligonucleotides enhances the systemic antitumor effects of radiotherapy and anti-PD-1 in metastatic non-small cell lung cancer. Cancer Immunol Res. (2023) 11:486–500. doi: 10.1158/2326-6066.CIR-22-0547 36700864 PMC10099280

[65] Kaneda MM Messer KS Ralainirina N Li H Leem CJ Gorjestani S . PI3Kγ is a molecular switch that controls immune suppression. Nature. (2016) 539:437–42. doi: 10.1038/nature19834 27642729 PMC5479689

[66] Yang L Li A Yu W Wang H Zhang L Wang D . Blockade of purine metabolism reverses macrophage immunosuppression and enhances anti-tumor immunity in non-small cell lung cancer. Drug Resist Update. (2025) 78:101175. doi: 10.1016/j.drup.2024.101175 39608215

[67] Chao MP Weissman IL Majeti R . The CD47-SIRPα pathway in cancer immune evasion and potential therapeutic implications. Curr Opin Immunol. (2012) 24:225–32. doi: 10.1016/j.coi.2012.01.010 22310103 PMC3319521

[68] Feng M Jiang W Kim BYS Zhang CC Fu YX Weissman IL . Phagocytosis checkpoints as new targets for cancer immunotherapy. Nat Rev Cancer. (2019) 19:568–86. doi: 10.1038/s41568-019-0183-z 31462760 PMC7002027

[69] Liang J Bi G Huang X Xu Z Huang Y Bian Y . CD24 is a promising immunotherapeutic target for enhancing efficacy of third-generation EGFR-TKIs on EGFR-mutated lung cancer. Cancer Commun (Lond). (2025) 45:1547–78. doi: 10.1002/cac2.70068 41084191 PMC12629863

[70] Fan Q Li J Qu X Liu Y Ren Y Zhang J . Selective depletion of tumor-associated macrophages with biomineralized erythrocyte nanoghosts to boost neoadjuvant radioimmunotherapy. ACS Nano. (2026) 20:5561–76. doi: 10.1021/acsnano.5c16031 41666246

[71] Liang Q Peng Y Zhao L Yang Z Chen X Li J . Pulmonary macrophage-targeted RNAi and mRNA co-delivery via SORT lipid nanoparticles enhances immunotherapy in lung cancer. J Control Release. (2026) 390:114519. doi: 10.1016/j.jconrel.2025.114519 41371503

[72] Chai D Wang J Lim JM Xie X Yu X Zhao D . Lipid nanoparticles deliver DNA-encoded biologics and induce potent protective immunity. Mol Cancer. (2025) 24:12. doi: 10.1186/s12943-024-02211-8 39806486 PMC11727718

[73] Chang LC Chin YC Wu PC Wei YF Wu HC Cheng TY . Polymeric nano-formulation of spectrum selective RTK inhibitor strengthens anti-cancer effects via immune remodeling by endoplasmic reticulum stress-modulating mitochondrial metabolism. Nano Today. (2024) 54:102070. doi: 10.1016/j.nantod.2023.10207042574925

[74] Klichinsky M Ruella M Shestova O Lu XM Best A Zeeman M . Human chimeric antigen receptor macrophages for cancer immunotherapy. Nat Biotechnol. (2020) 38:947–53. doi: 10.1038/s41587-020-0462-y 32361713 PMC7883632

[75] Liu Q Liu X Zhang H Jiang Y Shi Y Mu Q . Integrative multi-omics and machine learning reveals the spatial niche distribution and role of CYP27A1+ TAMs in immunotherapy response in non-small cell lung cancer. Front Immunol. (2026) 17:1782545. doi: 10.3389/fimmu.2026.1782545 41836417 PMC12979556

[76] Gao L Liu Y Zou J Deng F Liu Z Zhang Z . Deep scSTAR: leveraging deep learning for the extraction and enhancement of phenotype-associated features from single-cell RNA sequencing and spatial transcriptomics data. Brief Bioinform. (2025) 26:bbaf160. doi: 10.1093/bib/bbaf160 40315434 PMC12047704

[77] Liu C Li K Sui X Zhao T Zhang T Chen Z . Patient-derived tumor organoids combined with function-associated scRNA-seq for dissecting the local immune response of lung cancer. Adv Sci (Weinh). (2024) 11:e2400185. doi: 10.1002/advs.202400185 38896792 PMC11336893

[78] Kim GD Shin SI Sun P Lee JE Chung C Kang YE . Single-cell RNA sequencing of baseline PBMCs predicts ICI efficacy and irAE severity in patients with NSCLC. J Immunother Cancer. (2025) 13:e011636. doi: 10.1136/jitc-2025-011636 40404203 PMC12097017

[79] Wei J Zhuang Y Jiang C Chen L Yuan B Zhao Y . Cohort-based pan-cancer analysis and experimental studies reveal ISG15 gene as a novel biomarker for prognosis and immunotherapy efficacy prediction. Cancer Immunol Immunother. (2025) 74:168. doi: 10.1007/s00262-025-04026-y 40208307 PMC11985735

[80] Zhang X Liu F . Development and validation of a prognostic model for lung cancer based on machine learning and immune microenvironment analysis. J Cell Mol Med. (2025) 29:e70962. doi: 10.1111/jcmm.70962 41319095 PMC12665117

[81] Walters H . Senescent macrophages drive lung cancer and accumulate in aging. Nat Aging. (2023) 3:757. doi: 10.1038/s43587-023-00459-1 37414988

[82] Haston S Gonzalez-Gualda E Morsli S Ge J Reen V Calderwood A . Clearance of senescent macrophages ameliorates tumorigenesis in KRAS-driven lung cancer. Cancer Cell. (2023) 41:1242–1260.e6. doi: 10.1016/j.ccell.2023.05.004 37267953

[83] Qin L Liang T Zhu X Hu W Li B Wei M . Senescent macrophages and the lung cancer microenvironment: a new perspective on tumor immune evasion. Aging Dis. (2025) 16:3453–65. doi: 10.14336/AD.2024.1404 39751858 PMC12539527

[84] Zhang ZH Yin JZ Li W Xiao H Wang D Yang MH . Multiomics analysis reveals that senescent CXCL16+ macrophages promote lung adenocarcinoma progression through TGF-β signalling. J Transl Med. (2026) 24:306. doi: 10.1186/s12967-026-07766-2 41622242 PMC12954971

[85] Yu H Zhao M Li Q Zhang Y . Decoding macrophage heterogeneity in the pulmonary fibrosis lung cancer transition. Front Immunol. (2026) 17:1787094. doi: 10.3389/fimmu.2026.1787094 41939908 PMC13046558

[86] Huang H Teng G Zhou J Liu L Dong M Chen W . Single cell transcriptomic profiling uncovers shared inflammatory pathways and immune dysregulation underlying the asthma to lung cancer transition. Discov Oncol. (2026) 17:8. doi: 10.1007/s12672-025-04307-z 41441926 PMC12770138

[87] Colegio OR Chu NQ Szabo AL Chu T Rhebergen AM Jairam V . Functional polarization of tumour-associated macrophages by tumour-derived lactic acid. Nature. (2014) 513:559–63. doi: 10.1038/nature13490 25043024 PMC4301845

[88] Zhang D Tang Z Huang H Zhou G Cui C Weng Y . Metabolic regulation of gene expression by histone lactylation. Nature. (2019) 574:575–80. doi: 10.1038/s41586-019-1678-1 31645732 PMC6818755

[89] De Palma M Venneri MA Galli R Sergi Sergi L Politi LS Sampaolesi M . Tie2 identifies a hematopoietic lineage of proangiogenic monocytes required for tumor vessel formation and a mesenchymal population of pericyte progenitors. Cancer Cell. (2005) 8:211–26. doi: 10.1016/j.ccr.2005.08.002 16169466

[90] Arlauckas SP Garris CS Kohler RH Kitaoka M Cuccarese MF Yang KS . *In vivo* imaging reveals a tumor-associated macrophage-mediated resistance pathway in anti-PD-1 therapy. Sci Transl Med. (2017) 9:eaal3604. doi: 10.1126/scitranslmed.aal3604 28490665 PMC5734617

[91] Zhang Y He B Zou P Wu M Wei M Xu C . Targeted release of a bispecific fusion protein SIRPα/Siglec-10 by oncolytic adenovirus reinvigorates tumor-associated macrophages to improve therapeutic outcomes in solid tumors. J Immunother Cancer. (2025) 13:e010767. doi: 10.1136/jitc-2024-010767 40169285 PMC11962785

